# Rhizobacterial community structure in response to nitrogen addition varied between two Mollisols differing in soil organic carbon

**DOI:** 10.1038/s41598-018-30769-z

**Published:** 2018-08-16

**Authors:** Tengxiang Lian, Zhenhua Yu, Junjie Liu, Yansheng Li, Guanghua Wang, Xiaobing Liu, Stephen J. Herbert, Junjiang Wu, Jian Jin

**Affiliations:** 1 Key Laboratory of Mollisols Agroecology, Northeast Institute of Geography and Agroecology, Chinese Academy of Sciences, Harbin, 150081 China; 2 College of Agriculture, South China Agricultural University, Guangzhou, 510642 China; 3 Centre for AgriBioscience, La Trobe University, Melbourne Campus, Bundoora, VIC, 3086 Australia; 4 Stockbridge School of Agriculture, University of Massachusetts, Amherst, MA 01003 USA; 5 Soybean Research Institute of Heilongjiang Academy of Agricultural Sciences, Key Laboratory of Soybean Cultivation of Ministry of Agriculture P. R. China, Harbin, China

## Abstract

Excessive nitrogen (N) fertilizer input to agroecosystem fundamentally alters soil microbial properties and subsequent their ecofunctions such as carbon (C) sequestration and nutrient cycling in soil. However, between soils, the rhizobacterial community diversity and structure in response to N addition is not well understood, which is important to make proper N fertilization strategies to alleviate the negative impact of N addition on soil organic C and soil quality and maintain plant health in soils. Thus, a rhizo-box experiment was conducted with soybean grown in two soils, i.e. soil organic C (SOC)-poor and SOC-rich soil, supplied with three N rates in a range from 0 to 100 mg N kg^−1^. The rhizospheric soil was collected 50 days after sowing and MiSeq sequencing was deployed to analyze the rhizobacterial community structure. The results showed that increasing N addition significantly decreased the number of phylotype of rhizobacteria by 12.3%, and decreased Shannon index from 5.98 to 5.36 irrespective of soils. Compared to the SOC-rich soil, the increases in abundances of *Aquincola* affiliated to Proteobacteria, and *Streptomyces* affiliated to Actinobacteria were greater in the SOC-poor soil in response to N addition. An opposite trend was observed for *Ramlibacter* belong to Proteobacteria. These results suggest that N addition reduced the rhizobacterial diversity and its influence on rhizobacterial community structure was soil-specific.

## Introduction

Worldwide, large amounts of nitrogen (N) are being applied into farmland ecosystems with frequently more than 100 kg N ha^−1^ ^1^. The N input in China is even more excessive. The mean N application rate per crop has reached 155 kg N ha^−1^ with the total N input of 24 Mt year^−1^, which accounts for about 30% of the world’s total consumption^2,3^.

The N addition not only exerts impact on soil chemistry such as eutrophication and soil acidification^4,5^, but also soil biological properties including reductions in soil respiration and microbial biomass, and loss of biodiversity^5–7^. Recently, using high-throughput sequencing technologies to determine the prokaryotic community structure in a semi-arid Leymus chinensis steppe, Yao *et al*.^8^ demonstrated that N addition decreased the relative abundance of Acidobacteria, Verrucomicrobia and Chloroflexi, while increased the abundance of Proteobacteria and Bacteroidetes. In an agricultural system, Zhong *et al*.^9^ found that a N rate of 180 kg ha^−1^ per year was the threshold to reduce microbial activity with primary changes in abundances of Bacteroides, Fibrobacteres and Gemmatimonadetes in a wheat growing Anthrosol.

However, the effect of N addition on bacterial diversity is likely site-dependent. For example, Fierer *et al*.^10^ reported that N addition resulted in significant decrease in bacterial phylotype diversity in an agricultural field, but not in grassland. It is expected that different bacterial communities between soils may have different sensitivities to N addition, and the bacterial response may markedly affect the ecosystem function and stability, highlighting the importance of investigating the soil bacterial response to N addition in different soils.

Moreover, in crop-grown soils, the rhizosphere is the hotspot for biochemical processes in soil as labile root exudates boosts the abundance and activity of certain bacteria from soil reservoir (rhizobacteria)^11^. Nevertheless, how N addition interacts with the rhizobacterial community in different soils remain unknown. Specifying the bacterial response to N addition in the rhizosphere of crops is important for developing N-strategies in farming soils that consider microbial ecoservices such as soil carbon (C) dynamics and plant health.

Therefore, this study aimed at the comparison of rhizobacterial diversity and structure in response to N addition between two major farming Mollisols in Northest China. This soil region is the world’s fourth largest contiguous bodies of Mollisols which are fertile for crop production^12^. Since biogeographical distribution of bacterial communities varies in the Mollisol region, and this variation is greatly attributed to the soil organic C (SOC) distribution along the latitude of this region^13^, we expected that the impact of N addition on bacterial community would be different in Mollisols differing in SOC.

## Materials and Methods

### Experimental design

A randomized complete block design was used in this experiment, which consisted of three treatments with three replicates in each treatment. The treatments included (1) non-nitrogen control, (2) 25 mg N kg^−1^, and (3) 100 mg N kg^−1^ addition as urea. The soils used in this study were classified as Mollisols (USDA soil taxonomy) and collected from approximately 0.1 m depth in the tillage layer at two sites located in Jilin (43°20′N, 124°28′E) and Heilongjiang (48°17′N, 127°15′E) Provinces in northeast China^14^. The SOC-poor soil had an organic C content of 18 mg g^−1^ soil, total nitrogen of 1.7 mg g^−1^ soil, total potassium of 16 mg g^−1^ soil, available N of 100 μg g^−1^ soil, available K of 100 μg g^−1^ soil. The SOC-rich soil had an organic C content of 50 mg g^−1^ soil, total nitrogen of 3.7 mg g^−1^ soil, total potassium of 12 mg g^−1^ soil, available N of 266 μg g^−1^ soil, available K of 130 μg g^−1^ soil. The soil from each location was bulked, air-dried and sieved through a 2-mm sieve.

The soybean (*Glycine max* L. Merr.) cultivar Suinong 14 (Maturity Group 0) was used in this study. This cultivar has been widely grown over 2 million ha, with a total grain yield of 937 million kg in Northeast China since it was released in 1996^15^.

### Experimental Set-up

A rhizo-box experiment was performed at the glasshouse at the Northeast Institute of Geography and Agroecology, Chinese Academy of Sciences, Harbin, China. The rhizo-box was established referring to Jin *et al*.^16^. In brief, the Perspex-made rhizo-box (100 mm wide × 150 mm high × 10 mm thick) was filled with 100 g of sieved soil and was placed upright on top of a pot containing 2.5 kg of sterilized sand that supplies water for the plants (Fig. S1). Basal nutrients were applied at the following rates (mg kg^−1^): 219, KH_2_PO_4_; 167, CaCl_2_.2H_2_O; 43, MgSO_4_.7H_2_O; 9, Fe-EDTA; 6, ZnSO_4_; 5, CuSO_4_; 0.7, H_3_BO_3_; 6.7, MnSO_4_.H_2_O; 0.3, CoSO_4_.7H_2_O; and 0.2, Na_2_MoO_4_.2H_2_O. Nutrients were thoroughly mixed with the soil, and added again 25 days after sowing. Six seeds of uniform size were sown into each rhizo-box and were thinned to two on the 10th day after sowing. The greenhouse had a night-time temperature range of 16 of 20 °C and a daytime temperature range of 24 to 28 °C. Soil water content was maintained at 80 ± 5% of field water capacity by weighing and sucking water from sand. Field water capacity was estimated by matric suction.

At harvest (50 days after sowing), the rhizo-box was carefully opened, and the rhizosphere soil was recovered by gently shaking the roots into a polyethylene bag before being mixed thoroughly. The soil from each rhizo-box was separated into three parts. Approximately 2 g were placed in an autoclaved microcentrifuge tube (2 ml) and frozen immediately in liquid nitrogen. Soil samples were then stored at −80 °C for DNA extraction. About 50 g of fresh soil in the rhizo-box were used for the measurements of microbial biomass C (MBC), dissolved organic C (DOC), ammonium (NH_4_^+^) and nitrate (NO_3_^−^). The MBC in the soil was measured by the chloroform-extraction method^17^. The amount of organic C for the non-fumigated soil samples corresponds to dissolved organic C (DOC)^18^. A continuous flow analytical system (SKALAR SAN^++^, The Netherlands) was used to determine NH_4_^+^ and NO_3_^−^ ^19^. The rest of soil was air dried for pH, and total C and N measurements. The pH was determined using a Thermo Orion 720 pH meter in H_2_O (1:5 = w:v). The soil total C and N were assayed using an Elemental III analyzer (Hanau, Germany).

Plants were separated into shoot and root. The root was washed with tap water to remove adhering soil particles. Both shoots and roots were dried at 70 °C for 72 h, and then finely ground in a ball mill (Restol MM2000, Retsch, Haan, Germany). The total C and N contents of plant samples were measured by an Elementar III analyser (Hanau, Germany).

### DNA extraction

Soil DNA of each sample was extracted from 0.5 g of frozen soil using a Fast DNA SPIN Kit for Soil (QbiogeneInc., Carlsbad, CA, USA). The extracted DNA was dissolved in a TE (10 mM Tris-HCl, 1 mM EDTA, pH 8.0) buffer, and total DNA was quantified with a NanoDrop Spectrophotometer (Bio-Rad Laboratories, Inc.).

### Sequencing of amplicons

Bacterial 16S rRNA genes were amplified with the primers 515f (5′-GTGCCAGCMGCCGCGG-3′) and 907r (5′-CCGTCAATTCMTTTRAGTTT-3′)^20^. The PCR mixture (25 μL) contained 22.5 μL of Platinum PCR SuperMix (TaKaRa, Dalian, China), 1 µL of each primer (10 μM) and 10 ng of genomic DNA, and sterilized ultra-pure water was added to make a total volume of 25 μL. The amplification conditions were as follows: 95 °C for 10 min (initial denaturation); 28 cycles of 95 °C for 15 s, 60 °C for 10 s, and 72 °C for 20 s; and an extension at 72 °C for 10 min^21^. Each DNA sample was amplified in duplicate, and the duplicate PCR products were combined and purified using the Agarose Gel DNA purification kit (TaKaRa, Dalian, China). The PCR amplicons were then sequenced using the Illumina MiSeq platform according to the standard protocol at Majorbio Bio-Pharm Technology Co., Ltd., Shanghai, China.

### Statistical analyses

After sequencing was completed, the quality of all sequence reads was checked using the Quantitative Insights Into Microbial Ecology (QIIME) pipeline (version 1.17; http://qiime.org/). Any ambiguous bases were excluded from further analysis, such as removal of sequences <220 bp with ambiguous base ‘N’ and an average base quality score <20^22^. Using CD-HIT, sequences with similarities >97% were clustered into one operational taxonomic unit (OTU)^23^. Because the number of sequences for samples varied between 7,594 and 9,808, a randomly selected subset of 7,594 contigs was applied to each sample to align the survey variation (number of sequences analysed per sample). Phylotypes were identified using the Ribosomal Database Project (RDP) pyrosequencing pipeline (http://pyro.cme.msu.edu/). Regarding α diversity, the Chao and Ace estimators, and Shannon index were obtained using the MOTHUR program (http://www.mothur.org). The rarefaction coverage was calculated by 1 − *n*_1_/*N*, in which *n*_1_/*N* is the ratio of the sequence that appeared only once (*n*_1_) to the total number of sequences (*N*)^24^. Regarding β diversity, principal coordinates analysis (PCoA) was used to indicate patterns of similarity (Bray-Curtis similarity) in microbial community composition between treatments^25,26^. Mantel-test (based on Bray-Curtis distance between environmental variables and OTU matrices) were used to test whether two or more matrixes were statistically correlated. A canonical correspondence analysis (CCA) was deployed to indicate the association between the bacterial community composition and the soil biochemical characteristics. Permutation test for CCA was used to analyze the statistical significance for the overall CCA model and the first two canonical axes. The statistical analyses such as PCoA and CCA were performed using the vegan package of R version 3.1.2 for Windows^27^.

With Genstat 13 (VSN International, Hemel Hemspstead, UK), the analysis of variance (ANOVA)^28^ was performed to indicate the treatment effect on soil biochemical properties, indices of α diversity, and the relative abundance of the bacterial groups at the genus level. This was based on the least significant difference (LSD) at the significant level of *p* < 0.05. The *p* values that were associated with relative abundances of phyla and genera were adjusted for multiple testing with the procedure of False Discovery Rate described by Benjamini and Hochberg^29^. The adjusted *p* values were marked as *Q* values. The relative abundances of genera above 0.3% in the bacterial community with significant (*Q* < 0.05) response to treatments were presented in this study. All sequences have been deposited into the GenBank short-read archive SRP077674 (PRJNA327270).

## Results

### Plant growth and biochemical properties in the rhizosphere

Nitrogen addition did not affect shoot dry weight in either SOC-poor or SOC-rich soil. The average of shoot dry weight across treatments was 5.55 and 5.28 g/pot for the SOC-poor and SOC-rich soils, respectively (Table S1). A similar trend was found in root. There was no N × soil interaction on either shoot or root dry weight.

Nitrogen addition resulted in the increase in NH_4_^+^ concentration in the rhizosphere of soybean grown in both soils (Table 1). There was no difference in NO_3_^−^ concentration among N treatments in the SOC-poor, while the concentration decreased with the increase of N rate in the SOC-rich soil, contributing to a significant N × soil interaction (*p* < 0.05). Nitrogen addition did not alter total N, SOC and C/N, while significantly increased MBC, with the greater increase at the rate of 25 mg N kg^−1^, compared to 100 mg N kg^−1^. Compared to the non-N control, pH in the rhizosphere considerably decreased in the treatment of 25 mg N kg^−1^ of N addition, but not in 100 mg N kg^−1^.

**Table 1:** Nitrogen addition, soil and their interactive effects on NH_4_^+^, NO_3_^−^, total C, N, C/N, microbial biomass C (MBC) and pH in the rhizosphere of soybean grown in the soil organic C (SOC)-poor and SOC-rich soils with different rates of N addition. *p* values less than 0.05 were indicated in bold letters.

	SOC-poor soil			SOC-rich soil			*ANOVA* (*p* values)			
	0 mg N kg^−1^	25 mg N kg^−1^	100 mg N kg^−1^	0 mg N kg^−1^	25 mg N kg^−1^	100 mg N kg^−1^	LSD_0.05_	N	Soil	N× Soil
NH_4_^+^ mg/kg	103 ± 8.54	101 ± 6.34	122 ± 13.5	125 ± 0.45	127 ± 2.94	141 ± 1.13	17	**0.018**	**0.002**	0.836
NO_3_^−^ mg/kg	3.36 ± 0.11	3.12 ± 0.45	3.20 ± 0.11	5.08 ± 0.28	3.52 ± 0.34	3.24 ± 0.40	0.76	**0.007**	**0.007**	**0.020**
Available N mg/kg	105 ± 8.65	104 ± 6.79	125 ± 13.63	129 ± 0.17	130 ±± 2.60	145 ± 0.74	18	**0.023**	**0.001**	0.818
Total N g/kg	0.11 ± 0.004	0.10 ± 0.002	0.10 ± 0.01	0.38 ± 0.02	0.39 ± 0.01	0.38 ± 0.01	0.32	0.075	<**0.001**	0.622
Total C g/kg	1.54 ± 0.04	1.55 ± 0.04	1.57 ± 0.09	5.14 ± 0.24	5.35 ± 0.12	5.15 ± 0.14	0.32	0.499	<**0.001**	0.494
C/N	13.9 ± 0.92	14.9 ± 0.10	15.2 ± 0.56	13.5 ± 0.11	13.7 ± 0.1	13.6 ± 0.04	1.10	0.141	**0.007**	0.232
MBC mg/kg	366 ± 139	1819 ± 263	1481 ± 141	1364 ± 161	2771 ± 518	2001 ± 332	717	**0.001**	**0.003**	0.489
DOC	2657 ± 26	2639 ± 55	2146 ± 23	2679 ± 22	2023 ± 23	2623 ± 83	190	**0.002**	0.350	<**0.001**
pH	5.34 ± 0.03	4.9 ± 0.04	5.64 ± 0.07	5.44 ± 0.05	4.91 ± 0.05	5.48 ± 0.01	0.11	<**0.001**	0.480	**0.021**

Values are means ± standard error (n = 3).

### Rhizobacterial α diversity

The phylotype number was in a range of 844 to 979 across the treatments with coverage over 0.96. Compared to the non-N control, increasing N addition rate significantly decreased phylotype number, resulting in a decrease from 5.95 to 4.99, and 6.01 to 5.72 for the SOC-poor and SOC-rich soils, respectively, when 100 mg N kg^−1^ was added. There was no significant N× soil interaction on phylotype number. However, N addition did not significantly alter Ace and Chao indices with 1,112 and 1,111 on average, respectively. Shannon indices significantly decreased with the increase of N addition, and it occurred in both the SOC-poor and SOC-rich soils (Table 2).

**Table 2:** Summary of phylotype number, rarefaction coverage, Ace, Chao and Shannon indices in the rhizosphere of soybean grown in the soil organic C (SOC)-poor and SOC-rich soils with N supply regime. Values are means ± standard error (n = 3). Significant levels of main effects, i.e. N and soil, and their interactions were presented. *p* values less than 0.05 were indicated in bold letters.

	SOC-poor soil			SOC-rich soil			*ANOVA* (*p* values)			
	0 mg N kg^−1^	25 mg N kg^−1^	100 mg N kg^−1^	0 mg N kg^−1^	25 mg N kg^−1^	100 mg N kg^−1^	LSD_0.05_	N	Soil	N × Soil
Phylotype number	979 ± 12	891 ± 17	854 ± 32	956 ± 21	860 ± 30	844 ± 37	81	**0.020**	0.329	0.929
Coverage	0.97 ± 0.002	0.97 ± 0.001	0.96 ± 0.003	0.97 ± 0.003	0.97 ± 0.003	0.97 ± 0.002	0.01	0.327	**0.005**	0.293
Ace	1200 ± 34	1080 ± 49	1145 ± 70	1116 ± 57	1058 ± 82	1070 ± 61	50	0.074	0.054	0.647
Chao	1201 ± 40	1075 ± 59	1129 ± 87	1115 ± 66	1078 ± 67	1071 ± 61	116	0.122	0.150	0.495
Shannon	5.95 ± 0.07	5.14 ± 0.52	4.99 ± 0.68	6.01 ± 0.08	5.59 ± 0.27	5.72 ± 0.09	0.66	**0.020**	**0.036**	0.327

The number of phylotype was calculated by sequences at the 97% similarity level.

### Rhizobacteria β diversity

**Figure 1:**
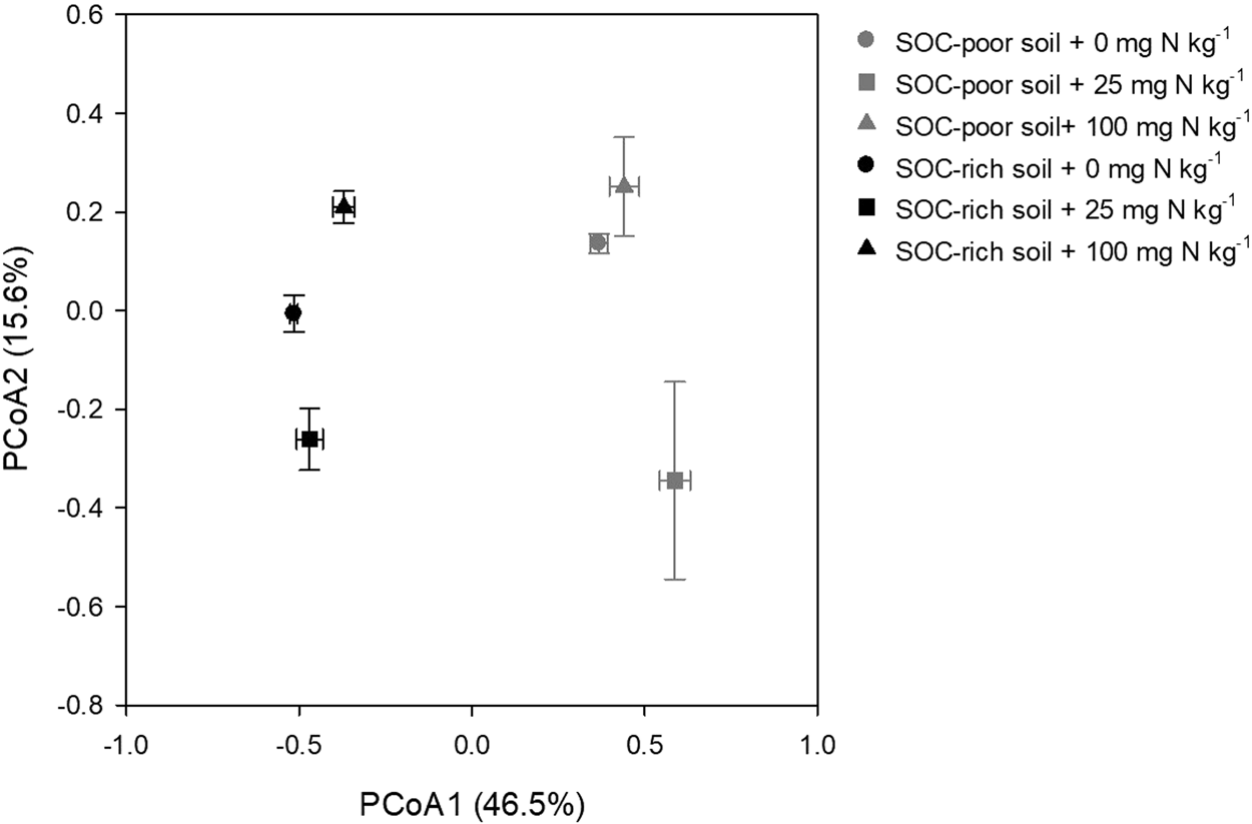
Principal coordinates analysis (PCoA) of bacterial community in the rhizosphere of soybean grown in the soil organic C (SOC)-poor and SOC-rich soils with different rates of N addition. Error bars on data points represent the standard error of the mean (n = 3).

Principal coordinate analysis (PCoA) showed that nitrogen fertilizer addition considerably altered the community structure in the rhizosphere of soybean. Moreover, N-induced change in the community composition varied between the two soils (*p* < 0.05) (Fig. 1). In particular, the separation of bacterial community in the N treatments from that in non-N treatment was greater in the SOC-poor soil compared with that in the SOC-rich soil.

Nitrogen addition and soil affected the abundances of 36 genera affiliated to 10 phyla (Tables 3 and S2). Briefly, in the phylum Proteobacteria, the abundance of *Nitrosomonadaceae-uncultured* significantly decreased from 2.48% in the non-N control to 1.62% in the 100 mg N kg^−1^ treatment in the SOC-poor soil and from 4.49% to 2.39% in the SOC-rich soil. Nitrogen addition significantly decreased the abundances of *Ramlibacter* in the SOC-poor soil, while opposite trend was found in the SOC-rich soil. Nitrogen application increased the abundance of *Aquincola* greater in the SOC-poor soil than the SOC-rich soil, contributing to a significant N× soil interaction (Table 3). In phylum Acidobacteria, genera *Blastocatella*, *RB41_norank* and *Acidobacteriaceae_(Subgroup 1)_uncultured* had lower abundances in N addition treatments compared to the non-N control. In phylum Actinobacteria, genus *Streptomyces* had a 3.5-fold increase in abundance in the SOC-poor soil when 25 mg N kg^−1^ was applied. However, only 2.3-fold of increase in abundance occurred in the SOC-rich soil when 100 mg N kg^−1^ applied. The abundances of *Roseiflexus*, *KD4-96_norank* and *Anaerolineaceae_uncultured* affiliated to Chloroflexi significantly decreased in the N addition treatments in both soils.

**Table 3:** The relative abundances of rhizobacteria at the genus level in the rhizosphere of soybean grown in the soil organic C (SOC)-poor and SOC-rich soils with different rates of N addition.

Phylum	Genus	SOC-poor soil			SOC-rich soil			ANOVA (*Q values*)			
		0 mg N kg^−1^	25 mg N kg^−1^	100 mg N kg^−1^	0 mg N kg^−1^	25 mg N kg^−1^	100 mg N kg^−1^	LSD_0.05_	N	Soil	N× Soil
Proteobacteria	*Sphingomonas*	4.30 ± 0.28	2.99 ± 1.88	2.53 ± 1.19	1.06 ± 0.31	1.46 ± 0.13	1.55 ± 0.47	1.68	0.539	**0.004**	0.401
	*Burkholderia*	1.97 ± 1.10	1.19 ± 0.34	0.82 ± 0.67	0.15 ± 0.04	0.53 ± 0.23	0.09 ± 0.03	0.98	0.256	**0.004**	0.428
	*Nitrosomonadaceae_uncultured*	2.48 ± 0.53	1.36 ± 0.80	1.62 ± 0.42	4.49 ± 0.51	2.54 ± 0.86	2.39 ± 0.42	1.09	**0.004**	<**0.001**	0.457
	*Ramlibacter*	1.80 ± 0.41	0.59 ± 0.37	1.19 ± 0.12	1.76 ± 0.23	1.73 ± 0.19	4.30 ± 1.08	1.58	**0.014**	**0.045**	**0.046**
	*Pseudolabrys*	1.41 ± 0.26	1.24 ± 0.71	1.07 ± 0.20	0.39 ± 0.08	1.13 ± 0.17	1.07 ± 0.22	0.61	0.427	0.055	**0.048**
	*Phenylobacterium*	0.97 ± 0.07	1.17 ± 0.80	1.55 ± 0.15	0.64 ± 0.09	0.68 ± 0.05	0.99 ± 0.41	0.67	0.181	**0.039**	0.929
	*Rhodopseudomonas*	1.07 ± 0.22	0.77 ± 0.18	0.74 ± 0.24	0.64 ± 0.02	0.44 ± 0.29	0.46 ± 0.08	0.35	0.113	**0.009**	0.927
	*Haliangium*	1.09 ± 0.27	0.85 ± 0.55	0.79 ± 0.09	0.62 ± 0.13	0.52 ± 0.15	0.47 ± 0.14	0.49	0.424	**0.024**	0.929
	*Mesorhizobium*	0.67 ± 0.09	0.67 ± 0.14	0.69 ± 0.15	0.14 ± 0.05	0.38 ± 0.18	0.32 ± 0.11	0.23	0.324	<**0.001**	0.550
	*Azospirillum*	0.25 ± 0.39	0.18 ± 0.30	1.98 ± 0.66	0.10 ± 0.06	0.03 ± 0.03	0.71 ± 0.29	0.63	<**0.001**	**0.016**	**0.046**
	*Novosphingobium*	0.63 ± 0.11	0.39 ± 0.56	0.81 ± 0.24	0.38 ± 0.11	0.23 ± 0.10	0.98 ± 0.13	0.47	**0.018**	0.576	0.635
	*Rhizomicrobium*	0.64 ± 0.06	0.51 ± 0.18	0.29 ± 0.14	0.25 ± 0.07	0.35 ± 0.09	0.11 ± 0.05	0.19	<**0.001**	<**0.001**	0.428
	*Aquincola*	1.81 ± 1.24	1.68 ± 2.70	11.1 ± 3.02	0.99 ± 0.27	0.11 ± 0.12	3.27 ± 1.46	3.26	**<0.001**	**0.006**	**0.025**
	*Comamonadaceae_unclassified*	0.92 ± 0.43	0.83 ± 0.47	2.09 ± 0.83	0.98 ± 0.11	0.79 ± 0.30	3.05 ± 0.73	0.95	<**0.001**	0.274	0.457
	*Rhodanobacter*	0.46 ± 0.12	0.80 ± 0.31	0.82 ± 0.23	0.40 ± 0.13	0.28 ± 0.14	0.27 ± 0.13	0.34	0.540	**0.004**	0.080
	*Rhizobium*	0.41 ± 0.13	0.11 ± 0.06	1.22 ± 0.68	0.31 ± 0.06	0.13 ± 0.08	0.65 ± 0.16	0.53	**0.004**	0.195	0.457
	*JG34-KF-161_norank*	0.58 ± 0.12	0.61 ± 0.14	0.57 ± 0.28	1.11 ± 0.25	0.94 ± 0.57	1.15 ± 0.28	0.55	0.869	**0.014**	0.916
	*Microvirga*	0.47 ± 0.03	0.31 ± 0.14	0.29 ± 0.16	0.33 ± 0.07	0.66 ± 0.15	0.86 ± 0.30	0.29	0.262	**0.012**	**0.025**
	*Caulobacteraceae_uncultured*	0.43 ± 0.02	0.31 ± 0.18	0.62 ± 0.21	0.54 ± 0.08	0.27 ± 0.01	0.45 ± 0.08	0.22	**0.023**	0.576	0.428
	*DA111_norank*	0.43 ± 0.01	0.52 ± 0.11	0.23 ± 0.13	0.20 ± 0.08	0.16 ± 0.05	0.16 ± 0.04	0.15	**0.043**	**<0.001**	**0.046**
	*Sphingomonadaceae_unclassified*	0.42 ± 0.03	0.11 ± 0.16	0.53 ± 0.18	0.16 ± 0.02	0.03 ± 0.02	0.36 ± 0.08	0.18	**<0.001**	**0.012**	0.623
	*AKYH478_norank*	0.47 ± 0.30	1.02 ± 0.12	0.35 ± 0.11	0.66 ± 0.21	0.61 ± 0.33	0.84 ± 0.11	0.39	0.199	0.464	**0.025**
	*Hyphomicrobium*	0.25 ± 0.14	0.14 ± 0.10	0.12 ± 0.02	0.00 ± 0.01	0.04 ± 0.02	0.01 ± 0.01	0.13	0.427	**<0.001**	0.457
	*TRA3-20_norank*	0.30 ± 0.07	0.38 ± 0.05	0.19 ± 0.13	0.47 ± 0.19	0.43 ± 0.19	0.22 ± 0.03	0.22	0.054	0.252	0.805
	*Myxococcales_uncultured*	0.36 ± 0.04	0.16 ± 0.09	0.20 ± 0.03	0.41 ± 0.13	0.24 ± 0.05	0.44 ± 0.08	0.14	**0.014**	**0.014**	0.387
Acidobacteria	*Subgroup_6_norank*	4.91 ± 1.20	1.76 ± 0.39	1.76 ± 0.48	4.21 ± 0.50	2.24 ± 1.08	2.36 ± 1.98	1.95	**0.007**	0.826	0.753
	*Acidobacteriaceae_(Subgroup_1)_uncultured*	2.09 ± 0.48	0.99 ± 0.26	1.14 ± 0.40	0.72 ± 0.24	0.50 ± 0.32	0.34 ± 0.18	0.59	**0.014**	**<0.001**	0.360
	*RB41_norank*	1.98 ± 0.36	1.01 ± 0.17	0.46 ± 0.19	2.63 ± 0.34	1.07 ± 0.76	1.01 ± 0.99	0.99	**<0.001**	0.184	0.831
	*Blastocatella*	1.56 ± 0.34	0.65 ± 0.06	0.76 ± 0.22	0.89 ± 0.27	0.49 ± 0.28	0.65 ± 0.46	0.53	**0.014**	0.067	0.457
	*ABS-19_norank*	0.86 ± 0.25	1.39 ± 0.27	0.61 ± 0.18	0.40 ± 0.21	0.57 ± 0.11	0.30 ± 0.06	0.34	**0.007**	**<0.001**	0.360
	*Telmatobacter*	0.41 ± 0.35	0.20 ± 0.09	0.22 ± 0.10	0.01 ± 0.01	0.00 ± 0.00	0.00 ± 0.00	0.27	0.472	**0.006**	0.713
	*Subgroup_4_norank*	0.38 ± 0.03	0.11 ± 0.04	0.10 ± 0.07	0.43 ± 0.15	0.25 ± 0.11	0.25 ± 0.18	0.20	**0.014**	0.073	0.884
	*Granulicella*	0.20 ± 0.13	0.17 ± 0.04	0.12 ± 0.07	0.03 ± 0.03	0.01 ± 0.02	0.00 ± 0.00	0.11	0.423	**<0.001**	0.923
	*Candidatus_Koribacter*	0.28 ± 0.05	0.15 ± 0.01	0.19 ± 0.05	0.07 ± 0.02	0.04 ± 0.02	0.07 ± 0.01	0.06	**0.012**	**<0.001**	**0.048**
Actinobacteria	*Streptomyces*	4.44 ± 1.41	15.5 ± 1.10	2.64 ± 0.44	1.29 ± 0.77	1.39 ± 0.34	3.02 ± 0.37	8.08	0.125	**0.039**	**0.048**
	*Gaiellales_uncultured*	1.91 ± 0.13	2.12 ± 0.49	1.17 ± 0.31	3.71 ± 0.54	4.83 ± 1.32	3.26 ± 0.54	1.19	**0.040**	**<0.001**	0.728
	*Arthrobacter*	1.28 ± 0.17	0.57 ± 0.15	1.11 ± 0.34	3.13 ± 0.47	2.05 ± 0.31	2.21 ± 0.26	0.04	**<0.001**	0.542	0.956
	*Actinoplanes*	0.79 ± 0.11	0.93 ± 0.22	0.45 ± 0.14	0.26 ± 0.07	0.18 ± 0.12	0.15 ± 0.09	0.24	**0.025**	**<0.001**	**0.048**
	*Acidimicrobiales_uncultured*	0.83 ± 0.07	0.84 ± 0.15	0.42 ± 0.23	1.20 ± 0.13	1.03 ± 0.39	0.79 ± 0.23	0.40	**0.035**	**0.023**	0.895
	*Blastococcus*	0.58 ± 0.04	0.83 ± 0.23	1.00 ± 0.30	0.67 ± 0.08	0.90 ± 0.51	1.33 ± 0.32	0.52	**0.044**	0.313	0.887
	*Patulibacter*	0.27 ± 0.11	0.58 ± 0.09	0.15 ± 0.07	0.38 ± 0.25	1.37 ± 0.22	0.00 ± 0.00	0.27	**<0.001**	**0.011**	**0.001**
	*Streptosporangiaceae_norank*	0.23 ± 0.19	0.11 ± 0.08	0.22 ± 0.12	0.01 ± 0.02	0.02 ± 0.03	0.03 ± 0.02	0.18	0.524	**0.011**	0.728
	*Gaiella*	0.13 ± 0.01	0.20 ± 0.04	0.11 ± 0.03	0.81 ± 0.06	0.80 ± 0.22	0.53 ± 0.04	0.30	**<0.001**	0.221	0.678
	*Microlunatus*	0.35 ± 0.04	0.22 ± 0.07	0.18 ± 0.05	0.78 ± 0.15	0.74 ± 0.43	0.75 ± 0.16	0.36	0.693	**<0.001**	0.929
Bacteroidetes	*Flavisolibacter*	0.94 ± 0.14	0.74 ± 0.32	0.73 ± 0.13	0.61 ± 0.11	0.86 ± 0.21	1.01 ± 0.11	0.33	0.693	0.845	**0.048**
	*Niastella*	0.65 ± 0.13	0.17 ± 0.07	0.86 ± 0.33	0.69 ± 0.46	0.30 ± 0.14	2.47 ± 1.31	1.05	**0.012**	0.075	0.069
Candidate_division_TM7	*Candidate_division_TM7_norank*	0.44 ± 0.32	0.28 ± 0.11	0.20 ± 0.08	2.62 ± 1.21	13.6 ± 2.91	0.01 ± 0.01	3.99	**<0.001**	**<0.001**	**0.001**
Chloroflexi	*KD4-96_norank*	1.59 ± 0.43	0.83 ± 0.06	1.48 ± 0.76	2.87 ± 0.13	1.62 ± 0.78	1.51 ± 0.73	1.01	**0.044**	**0.039**	0.450
	*Roseiflexus*	0.50 ± 0.04	0.23 ± 0.02	0.27 ± 0.06	0.76 ± 0.04	0.42 ± 0.11	0.66 ± 0.13	0.24	**<0.001**	**0.014**	0.680
	*TK10_norank*	1.35 ± 0.21	1.08 ± 0.08	0.65 ± 0.23	1.08 ± 0.13	0.90 ± 0.44	0.73 ± 0.03	0.41	**0.016**	0.313	0.671
	*Anaerolineaceae_uncultured*	1.55 ± 0.50	0.69 ± 0.20	0.76 ± 0.30	2.20 ± 0.86	1.40 ± 0.25	1.26 ± 0.66	0.92	**0.040**	**0.043**	0.956
	*C0119_norank*	0.91 ± 0.07	0.57 ± 0.05	0.43 ± 0.05	0.54 ± 0.11	0.48 ± 0.14	0.40 ± 0.06	0.15	**<0.001**	**0.004**	**0.025**
Cyanobacteria	*Cyanobacteria_norank*	0.36 ± 0.29	0.22 ± 0.07	4.86 ± 3.29	0.32 ± 0.20	0.28 ± 0.16	1.76 ± 1.65	2.69	**0.014**	0.223	0.428
Firmicutes	*Asteroleplasma*	0.30 ± 0.17	2.78 ± 0.98	0.38 ± 0.09	0.04 ± 0.04	0.14 ± 0.21	0.00 ± 0.00	0.74	<0.001	**<0.001**	**0.001**
Gemmatimonadetes	*Gemmatimonas*	0.38 ± 0.16	0.30 ± 0.01	0.19 ± 0.05	0.54 ± 0.27	0.97 ± 0.46	0.57 ± 0.07	0.41	0.229	**0.009**	0.450
Nitrospirae	*Nitrospira*	0.47 ± 0.14	0.13 ± 0.02	0.11 ± 0.06	0.62 ± 0.15	0.29 ± 0.13	0.28 ± 0.07	0.19	**<0.001**	**0.014**	0.986
Verrucomicrobia	*DA101_soil_group_norank*	0.49 ± 0.38	0.17 ± 0.05	0.16 ± 0.03	1.77 ± 0.43	1.05 ± 0.96	1.37 ± 1.42	1.31	0.524	**0.014**	0.929
WCHB1-60	*WCHB1-60_norank*	1.46 ± 0.60	1.83 ± 1.20	0.29 ± 0.09	0.19 ± 0.17	0.96 ± 0.61	0.04 ± 0.01	1.08	**0.028**	**0.029**	0.623

Genera with abundance above 0.3% and significance (*Q* < 0.05) on one of main effects, i.e. N and soil, or their interaction were presented. Values are means ± standard error (n = 3). *Q* values less than 0.05 were indicated in bold letters. The *Q* values indicate the *p* values have been corrected using False Discovery Rate.

**Figure 2:**
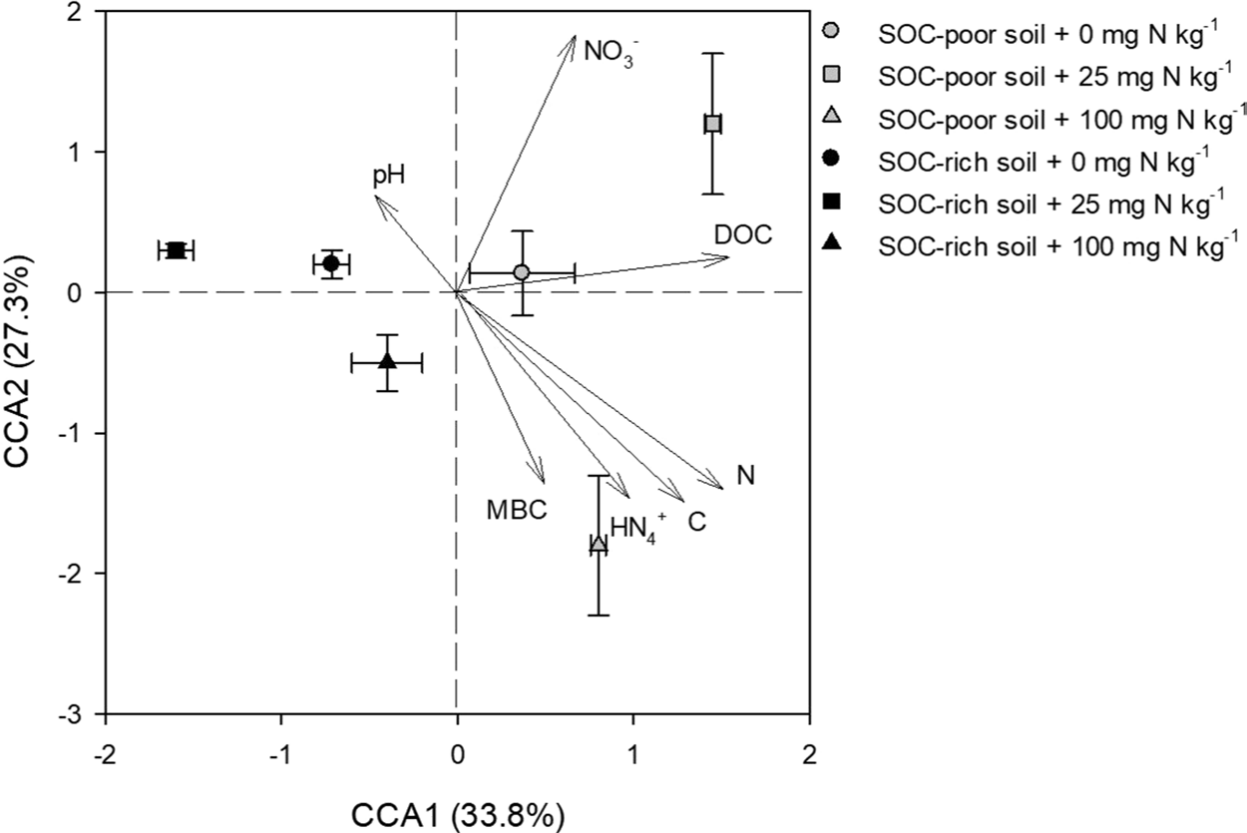
Canonical correspondence analysis (CCA) considering the relative abundance of bacteria at the operational taxonomic units (OTUs) level and soil total C and N, concentrations of NH_4_^+^ and NO_3_^−^ and pH in the rhizosphere of soybean grown in soil organic C (SOC)-poor and SOC-rich soils with different rates of N addition. The significance level of *p* < 0.001 was for the overall CCA model, and for the X and Y axes as well with 999 of permutations. Error bars on data points represent the standard error of the mean (n = 3).

Based on 999 permutations, the CCA analysis indicated that the rhizobacterial community composition was significantly associated (*p* < 0.001) with the biochemical variables that were mainly total C, total N, available N and DOC (Fig. 2). In particular, total C (r = 0.216; *p* < 0.05) and N (r = 0.222; *p* < 0.05), NO_3_^−^ (r = 0.341; *p* < 0.05), and DOC (r = 0.251; *p* < 0.05) appeared to be strongly linked to microbial community composition (Table S3).

## Discussion

Increasing N addition greatly decreased the diversity of rhizobacteria in both soils. It was evident that the phylogenetic number and Shannon index significantly decreased in response to N addition (Table 2). Similar results were observed by previous studies in Arctic tundra soil^30^, maize-grown loamy soil^7^, C3 rhizome grass-grown loamy sand^8^ and wheat-grown silty soil^31^. The reduction in the bacterial diversity was expected to be associated with soil acidification and consequent accumulation of toxins leading to a negative impact on soil microbial diversity^32,33^. However, it was not the case in this study, as pH did not change significantly at 100 mg N kg^−1^ input compared to the non-N control (Tables 1 and 3). The direct effect of N availability of in the soil was mainly responsible for the decrease of bacterial diversity. As majority of microbes in soil are k strategists and in a dormant state^11^, more available N in the rhizosphere under N addition in this study (Table 1) favored the relative abundance of those bacteria with a fast growth rate (r strategists) such as Proteobacteria, Actinobacteria, Bacteroidetes and Firmicutes^7^. Thus, the k strategists such as Chloroflexi and Acidobacteria were likely suppressed (Table S2), leading to decline in the microbial diversity. Fan *et al*.^34^ investigated bacterial communities in the nutrient-rich niche of wheat rhizoshphere across 800,000 km^2^ of north China plain, and found consistent decrease in phylogenetic diversity compared to that in the bulk soil, which supports the view of this point. However, whether the decrease in bacterial diversity in response to N addition was associated with soil quality and consequent plant N uptake remains unknown. Chemically, low soil pH inhibits the uptake of ammonium by soybean plants^35^. Therefore, increasing soil pH may be an effective strategy to improve soil quality and nutrient uptake.

Nitrogen addition affected the abundances of a number of genera across several phyla, in which most of genera were associated with N metabolism. For example, the increase in the abundance of *Cyanobacteria_norank* under N addition may be due to its great demand for N, as approximately 10% of the dry weight of cyanobacterial cells compromise of N, and nitrate and ammonium are virtually universal sources of N for *Cyanobacteria*^36^. Interestingly, *Nitrosomonadaceae* are able to oxidize ammonia to nitrate^37^, but its abundance decreased with the decrease of NO_3_^−^ concentration in response to N addition (Tables 1 and 3), reflecting that there were other factors limiting the growth of *Nitrosomonadaceae*, such as pH change, intermediary products during the oxidization. In addition, the N-suppressed genera, *Acidobacteriaceae_(Subgroup 1)_uncultured* and *RB41_norank* have been reported with capability of degrading polysaccharide^38^, but whether N addition inhibits the decomposition of recalcitrant organic matter requires further study.

The impact of N addition on the abundances of a number of major genera was dependent of soil type, and these responses may be associated with C cycling and plant health. In terms of C cycling,* Aquincola* are capable to utilize the mannose, maltose and butyrate^39^, under N addition conditions. The greater increase in its abundance in the SOC-poor soil than the SOC-rich soil may accelerate the decomposition of root exudates when the amount of N was amended. Regarding plant health, some *Streptomyces* sepecies can cause plant diseases. *Streptomyces scabies* (syn. scabiei) and *Streptomyces europaeiscabiei*, for instance, are common scab causing pathogen and distributed worldwide^40^. However, a number of genomes in *Streptomyces* may have the function of producing antibiotics and siderophore that may benefit plant health^41^. Thus, in this study, the ecofunction for greater N-induced increase in the abundance of *Streptomyces* in the SOC-poor soil requires specific investigation regarding the association of such microbes with plant health under N addition.

## Conclusions

Nitrogen addition decreased the diversity of rhizobacteria with significant decreases in the number of phylotype and Shannon index. The impact of N addition on the rhizobacterial community structure was soil-specific with greater increases in *Aquincola* affiliated to Proteobacteria, and *Streptomyces* affiliated to Actinobacteria in the SOC-poor soil compared to the SOC-rich soil, and an opposite trend for *Ramlibacter* belong to Proteobacteria. These changes may contribute to soil organic C turnover and plant pathogen under N addition, implying that N addition may mediate plant-soil-microbe relations in term of soil quality and plant health.

## Electronic supplementary material

Supplementary Information
