# Supplementary material for: Rhizobacterial community structure in response to nitrogen addition varied between two Mollisols differing in soil organic carbon
Source: Sci Rep. 2018 Aug 16;8:12280. doi: 10.1038/s41598-018-30769-z (PMC6095926; doi:10.1038/s41598-018-30769-z)
Supplement: Supplementary file 1 — Supplementary Information [file 41598_2018_30769_MOESM1_ESM.docx]

**Rhizobacterial community structure in response to nitrogen addition varied between two Mollisols differing in soil organic carbon**

Tengxiang Lian^1, 2^ · Zhenhua Yu^1^· Junjie Liu^1^ · Yansheng Li^1^· Guanghua Wang^1^· Xiaobing Liu^1^ ·Stephen J Herbert^4^ Junjiang Wu^5^ Jian Jin^1, 3 *^

^1.^ *Key Laboratory of Mollisols Agroecology, Northeast Institute of Geography and Agroecology, Chinese Academy of Sciences, Harbin 150081, China;*

^2.^ *College of Agriculture, South China Agricultural University, Guangzhou 510642, China*

^3.^ *Centre for AgriBioscience, La Trobe University, Melbourne Campus, Bundoora, VIC 3086, Australia.*

^4.^ *Stockbridge School of Agriculture, University of Massachusetts, Amherst, MA 01003, USA*

*^5.^ Soybean Research Institute of Heilongjiang Academy of Agricultural Sciences, Key Laboratory of Soybean Cultivation of Ministry of Agriculture P. R. China, Harbin, China*

^*^ Corresponding author at: 138 Haping Road, Harbin, 150081, China.

*E*-*mail addresses*: [jinjian29@hotmail.com](mailto:jinjian29@hotmail.com) (J. Jin).

**Figure S1** The schematic diagram of rhizo-box system. The Perspex-made compartment was filled with 100 g of soil and was placed upright on top of a pot containing 2.5 kg of sand supplying water for the plants.

**Table S1** Nitrogen addition, soil and their interactive effects on the shoot and root biomass of soybean grown in the soil organic C (SOC)-poor and SOC-rich Mollisols with different rates of N addition. Values are means ± standard error (n=3). *p* values less than 0.05 were indicated in bold letters.

|  |  | SOC-poor soil |  |  | SOC-rich soil |  |  | *ANOVA* (*p* values) | | |  |
| --- | --- | --- | --- | --- | --- | --- | --- | --- | --- | --- | --- |
|  | 0 mg N kg^-1^ | 25 mg N kg^-1^ | 100 mg N kg^-1^ | 0 mg N kg^-1^ | 25 mg N kg^-1^ | 100 mg N kg^-1^ | LSD_0.05_ | N | Soil | N×Soil | |
| Shoot (g/pot) | 5.06±1.17 | 5.22±1.00 | 6.37±1.67 | 4.49±1.06 | 5.83±1.25 | 5.52±1.00 | 1.43 | 0.074 | 0.509 | 0.308 | |
| Root (g/pot) | 1.17±0.87 | 1.00±0.53 | 1.67±0.76 | 1.06±0.55 | 1.25±0.77 | 1.00±0.74 | 0.84 | 0.182 | **0.024** | 0.160 | |

**Table S2** Relative abundances of rhizobacteria at the phylum level in the rhizosphere of soybean grown in soil organic C (SOC)-poor and SOC-rich Mollisols with different rates of N addition. Significant levels of main effects, i.e. N and soil, and their interactions were presented. Values are means (± standard error) (n=3). *Q* values less than 0.05 were indicated in bold letters. The *Q* values indicate the *p* values have been corrected using False Discovery Rate.

|  |  | SOC-poor soil |  |  | SOC-rich soil |  |  | *ANOVA* (*Q* values) | | |
| --- | --- | --- | --- | --- | --- | --- | --- | --- | --- | --- |
| phylum | 0 mg N kg^-1^ | 25 mg N kg^-1^ | 100 mg N kg^-1^ | 0 mg N kg^-1^ | 25 mg N kg^-1^ | 100 mg N kg^-1^ | LSD_0.05_ | N | Soil | N×Soil |
| Proteobacteria | 38.6±3.44 | 29.1±14.9 | 57.0±6.44 | 31.4±2.63 | 27.3±3.6 | 44.7±7.89 | 13.8 | **0.001** | 0.124 | 0.812 |
| Actinobacteria | 16.3±1.09 | 28.8±10.9 | 13.3±3.34 | 21.8±2.11 | 22.3±7.08 | 19.7±1.47 | 9.94 | 0.058 | 0.495 | 0.673 |
| Acidobacteria | 16.0±1.75 | 9.31±0.20 | 7.10±1.96 | 12.8±1.29 | 8.19±2.54 | 7.71±3.67 | 3.88 | **0.001** | 0.312 | 0.673 |
| Chloroflexi | 9.46±0.65 | 6.20±0.88 | 5.50±1.81 | 10.8±1.02 | 7.36±2.36 | 7.32±2.04 | 2.83 | **0.008** | 0.124 | 0.935 |
| Candidate_division_TM7 | 4.84±1.42 | 13.7±11.4 | 2.02±0.24 | 7.37±4.18 | 20.0±8.01 | 2.64±1.90 | 10.7 | **0.009** | 0.318 | 0.873 |
| Bacteroidetes | 3.83±0.30 | 2.28±1.11 | 3.99±1.34 | 3.98±0.97 | 3.36±0.84 | 5.99±1.29 | 1.84 | **0.022** | 0.106 | 0.673 |
| Gemmatimonadetes | 3.94±0.59 | 2.72±0.78 | 2.21±1.00 | 4.81±0.20 | 5.76±1.86 | 4.49±0.75 | 1.78 | 0.221 | **0.001** | 0.673 |
| Firmicutes | 1.30±0.39 | 3.54±1.00 | 1.42±0.10 | 0.39±0.11 | 0.73±0.27 | 1.27±0.46 | 0.88 | **0.007** | **0.001** | **0.022** |
| Verrucomicrobia | 1.13±0.75 | 0.50±0.14 | 0.62±0.00 | 2.67±0.49 | 1.64±1.20 | 1.79±1.65 | 1.62 | 0.495 | **0.044** | 0.935 |
| WCHB1-60 | 1.46±0.60 | 1.83±1.20 | 0.29±0.09 | 0.19±0.17 | 0.96±0.61 | 0.04±0.01 | 1.08 | **0.022** | **0.044** | 0.673 |
| others | 3.28±0.23 | 2.03±0.09 | 6.54±0.63 | 3.74±0.22 | 2.42±0.17 | 4.36±0.48 | 3.05 | 0.205 | 0.208 | 0.813 |

| Biochemical variables | r | Significance |
| --- | --- | --- |
| N | 0.222 | < 0.050 |
| C | 0.216 | < 0.050 |
| pH | 0.010 | 0.401 |
| NH_4_^+^ | 0.114 | 0.135 |
| NO_3_^-^ | 0.341 | < 0.050 |
| C/N | 0.063 | 0.255 |
| MBC | 0.008 | 0.460 |
| DOC | 0.257 | < 0.050 |

**Table S3** The significance on the association of rhizobacterial community composition with soil biochemical variables based on the mantel test with 999 permutations. MBC and DOC represent microbial biomass C and dissolved organic C in soil, respectively.
